# The protective effect of manganese superoxide dismutase from thermophilic bacterium HB27 on hydrochloric acid-induced chemical cystitis in rats

**DOI:** 10.1007/s11255-021-03054-8

**Published:** 2021-11-16

**Authors:** Nai-wen Chen, Jin-lai Gao, Hai-long Li, Hong Xu, Ling-feng Wu, Fan-guo Meng, Wei Chen, Yi-fang Cao, Wen-hua Xie, Xiao-qin Zhang, Shi-hui Liu, Jing Jin, Yi He, Jian-wei Lv

**Affiliations:** 1 Department of Urology, The Affiliated Hospital of Jiaxing University, Jiaxing, 314001 Zhejiang China; 2 Department of Pharmacology, College of Medical, Jiaxing University, Jiaxing, Zhejiang 314001 People’s Republic of China; 3 Redox Medical Center for Public Health, Medical College of Soochow University, Suzhou, 215123 Jiangsu China; 4 Department of Pharmacy, College of Medical, Jiaxing University, Jiaxing, Zhejiang 314001 People’s Republic of China; 5 Department of Urology, Renji Hospital, School of Medicine, Shanghai Jiaotong University, Shanghai, 200127 People’s Republic of China

**Keywords:** Cystitis, Bladder pain, Superoxide dismutase, Thermophilic bacterium

## Abstract

**Purpose:**

To evaluate the effects of manganese superoxide dismutase (Mn-SOD) from thermophilic bacterium HB27 (name as Tt-SOD) on chemical cystitis.

**Methods:**

Control and experimental rats were infused by intravesical saline or hydrochloric acid (HCl) on the first day of the experiments. Saline, sodium hyaluronate (SH) or Tt-SOD were infused intravesically once a day for three consequent days. On the fifth day, the rats were weighted and sacrificed following a pain threshold test. The bladder was harvested for histological and biochemical analyses.

**Results:**

Tt-SOD could reduce the bladder index, infiltration of inflammatory cells in tissues, serum inflammatory factors and SOD levels, *mRNA* expression of inflammatory factors in tissues, and increase perineal mechanical pain threshold and serum MDA and ROS levels in HCl-induced chemical cystitis. Furthermore, Tt-SOD alleviated inflammation and oxidative stress by the negative regulation of the NF-κB p65 and p38 MAPK signaling pathway.

**Conclusions:**

Intravesical instillation of Tt-SOD provides protective effects against HCl-induced cystitis.

## Introduction

Chemotherapy is recognized as the most common treatment for patients with bladder cancer, and chemical cystitis is the most common complication following chemotherapy. Such treatment of chemical cystitis is often tedious, complex, cumbersome, and time consuming. If required, additional surgical procedures are performed: bladder augmentation or ureteral replantation. This caused distress, depression, and in some instances lead to patients refusing further chemotherapy, thereby compromising their treatment plan. Chemical cystitis is mainly caused by harmful chemical substance or metabolite that enters bladder, which is characterized by bladder mucosal inflammation and damage. Bladder mucosa consists of the urothelium, basement membrane and underlying lamina propria. It is located in a very thin layer of bladder transitional epithelial tissue and plays an important role in defending against the invasion of harmful factors [1]. Bladder mucosa can prevent circulation of the urine with high permeability solutes as well as pathogen invasion into the urinary tissue and into the blood stream. Concomitantly, it can secrete inflammatory mediators, neurotransmitters and can also be used as a sensory and external signal to confer tissue protection [2, 3]. In the present study, a rat model of cystitis was established by intravesical instillation of HCl that results in bladder mucosa damage. This model has been successfully applied in earlier experimental studies of cystitis model, resulting in abnormal urination and histologically observable chemical cystitis in rats [4]. Furthermore, it has been reported that a large amount of ROS is produced during cystitis [5].

ROS is mainly generated by mitochondria, mostly from inflammatory cells, such as neutrophils [6]. In addition, once activated, these inflammatory cells will produce ROS through the myeloperoxidase (MPO) pathway [7]. At low extracellular levels, ROS species are well known for playing a regulatory role in signaling [8]. However, at high levels, ROS can cause oxidative stress through lipid peroxidation, denaturation of proteins and DNA mutation [9]. Oxidative stress is a common underlying process associated with a variety of disorders, such as metabolic syndrome, chronic inflammation and even cancer [10]. To protect against oxidative stress, the body's antioxidant system can scavenge excess ROS to defend the cell from damage. There are two types of antioxidant systems in the human body, those that involve antioxidant enzymes and those that are non-enzymatic [11]. It has been previously shown that antioxidant enzymes can relieve bladder mucosal damage caused by cystitis by maintaining the activity of antioxidant enzymes and rebalancing the redox state [12]. As an antioxidant enzyme, SOD can catalyze the metabolism of excess ROS to hydrogen peroxide and oxygen and plays an important role in defending the organism against the toxic effects of oxygen [13]. To date, the family of SOD enzymes includes the Cu/Zn-SOD, Fe-SOD, Mn-SOD and Ni-SOD, which maintain oxygen free radical balance by metabolizing excess ROS to form oxygen and hydrogen peroxide [14, 15]. It has been reported that inflammatory cells produce high levels of ROS on bladder outlet obstruction, bladder ischemia–reperfusion injury and cystitis in animal models [16–19]. Although several studies have revealed that SOD can eliminate excess ROS in tissues, its therapeutic effects are still not satisfactory, probably due to its instability, high-temperature resistance, acid and alkali resistance, and resistance to digestive enzymes [20].

In the present study, a Mn-SOD was used from Thermus thermophilus HB27 (denoted as Tt-SOD), which has a precise active center structure, thermodynamic stability and kinetic inertia. For example, Tt-SOD exhibits superior stability and activity under high-temperature conditions, high acidity and alkalinity and several types of proteases, such as pepsin and trypsin. In addition, Tt-SOD overcomes the shortcomings of existing related products, which have a high application value. A previous study has demonstrated that inflammatory bowel disease (IBD) is attenuated by treatment with Tt-SOD, which is possibly due to decreased SOD activity and attenuated neutrophil infiltration in IBD animal models [21]. The present study aimed to investigate the protective effect of Tt-SOD in HCl-induced chemical cystitis.

## Materials and methods

### Reagents

The following reagents were purchased from the indicated companies: Tt-SOD(REDOX PHARMATECH CO., LTD, Hangzhou, China); Sodium hyaluronate (SH) (Solarbio, Beijing, China); IL-1β, IL-6 and TNF-α ELISA Kits (R&D Systems, Inc., Minneapolis, USA); SOD and MDA Kits (Nanjing Jiancheng Bioengineering Institute, NanJing, China); Total RNA Kit (Tiangen Biotech, China), and a reverse transcription polymerase chain reaction (RT-PCR) kit (Bio-Rad, Hercules, CA, USA); ROS Fluorescent Probe -DHE Kit (Vigorous Biotechnology, Beijing, China); Anti-phos-p65 (Ser536) antibody(3033), Anti-p65 antibody (8242), Anti-p38 antibody (6390), Anti-phos-p38(Thr180/Tyr182) (4511), Anti-IκBα antibody(4812) (Cell Signaling Technology, Boston, USA).

### Animals

The experiments were carried out in female Sprague Dawley rats (200–250 g). The animals were kept in the laboratory and maintained at 25 ± 3 °C, with a 12 h light and 12 h darkness cycle. They were provided free access to food and water.

### Method of drug administration

This method refers to the previously published protocol of Konkol et al. [22]. The animals were anesthetized with 4% chloral hydrate (4% chloral hydrate/rat body weight = 1 ml/100 g). Subsequently, a sterile polyethylene catheter (PE10) was inserted into the urethra for continuous bladder irrigation with 0.9% saline. Bladders were emptied and washed with 0.9% saline three times before drug instillation. Drug (Saline, SH or Tt-SOD) was then instilled into the bladder via the PE10 and kept for 30 min prior to rinsing three times with 0.9% saline. Finally, bladders were emptied by catheterization.

### Experimental design

The rats were randomly assigned to one of the following treatment groups:

Group 1 (Sham group, *N* = 6): Rat bladders were treated only with saline once a day for 4 consecutive days. Saline (0.25 ml) was injected into the rat bladder for 30 min on days 2, 3 and 4. The pain threshold test was performed on day 5.

Group 2 (HCl group, *N* = 6): Rat bladders were washed and emptied with saline three times before and after drug administration. The tissues were infused with HCl (0.25 ml, 0.1 mol/l) for 90 s to induce chemical cystitis by a sterile polyethylene catheter (PE10) on day 1. Then, saline (0.25 ml) was injected into the rat bladder for 30 min on days 2, 3 and 4. Pain threshold test was performed on day 5.

Group 3 (SH group, *N* = 6): Rat bladders were washed and emptied with saline three times before and after drug administration. The tissues were infused with HCl (0.25 ml, 0.1 mol/l) for 90 s to induce chemical cystitis by a sterile polyethylene catheter (PE10) on day 1. Then, SH (0.25 ml, 0.8 g/l) was injected into the rat bladder for 30 min on days 2, 3 and 4. Pain threshold test was performed on day 5.

Group 4 (Tt-SOD 100 U/ml group, *N* = 6): Rat bladders were treated with washed and emptied with saline three times before and after drug administration. The tissues were infused with HCl (0.25 ml, 0.1 mol/l) for 90 s to induce chemical cystitis by a sterile polyethylene catheter (PE10) on day 1. Tt-SOD (0.25 ml, 100 U/l) was injected into the rat bladder for 30 min on days 2, 3 and 4. The pain threshold test was performed on day 5.

Group 5 (Tt-SOD 300 U/ml group, *N* = 6): Only the dosing concentration of Tt-SOD was different and the other operations were the same as group 4.

Group 6 (Tt-SOD 1000 U/ml group, *N* = 6): Only the dosing concentration of Tt-SOD was different and the other operations were the same as group 4.

The animals were sacrificed by administering an overdose of isoflurane and their bladders were harvested, 24 h following the *pain threshold assessment.* Subsequently, the bladder was divided equally into three parts. One part of the bladder was stored at − 80 °C until further biochemical analysis, whereas the other parts were used for histological measurements.

### Pain threshold assessment

The assessment of the bladder-related pain was conducted as determined by Wang et al. and Jia-Liang Chen et al., which examined pain assessment in rats [23, 24]. The animals were placed in a metal mesh on a plastic cage for 30 min prior to testing, in a quiet environment, at stable room temperature. Since direct assessment of bladder-related pain is often difficult, we measured the perineal mechanical threshold as a substitute for bladder-related pain in the HCl-induced cystitis model. von Frey monofilaments (BME-404; Institute of Biological Medicine, Academy of Medical Science, Beijing, China) were used to measure the stimulated values regarding the perineal mechanical threshold. Abrupt retraction of abdomen, immediate licking, jumping and scratching of the site of application were considered as the mechanical threshold. The stimulated values were averaged following five consecutive tests and stimulation site was between the anus and the external urethral orifice. All the tests were performed by researchers who were blinded to the group.

### Analysis of inflammatory cytokines

Prior to sacrificing, 3 ml blood samples were collected from the heart of each animal under general anesthesia. Subsequently, the blood was centrifuged at 1000 × *g* for 20 min and the serum was obtained. The serum was used for the detection of inflammatory mediators (IL-1β, IL-6 and TNF-α) by ELISA according to the manufacturer's instructions and previous studies.(Yang S, Traore Y, Jimenez C, Ho EA. Autophagy induction and PDGFR-β knockdown by siRNA-encapsulated nanoparticles reduce Chlamydia trachomatis infection. Sci Rep 2019; 9: 1306.)

### Assessment of bladder index

Body and bladder weight (wet weight) was weighed on the date of euthanasia and the bladder index was calculated as the proportion of bladder weight (mg) to body weight (g)*100% [25].

### Determination of SOD and MDA content

The bladder samples were washed repeatedly with sterile normal saline to remove excess blood. Following removal of excess water with paper towels, the samples were weighed and sterile normal saline (0.9 ml/100 mg) was added to prepare the tissue homogenate by a tissue homogenizer. The homogenates were centrifuged at 5000 r.p.m. at 4 °C for 10 min and the supernatants were collected to measure the expression of MDA and SOD activity by Elisa kit (Nanjing Jiancheng Bioengineering Institute, Nanjing, China).

### Histological measurements

Bladder tissue was fixed in 4% paraformaldehyde fixative solution in the presence of 30% sucrose gradient dehydration solution for 48 h and subsequently used to produce frozen sections and paraffin sections.

### ROS fluorescent probe-DHE

Dihydroethidium (DHE) staining was performed as previously described [26]. In general, bladder tissue was fixed in 4% paraformaldehyde fixative solution in the presence of 30% sucrose gradient dehydration solution for 48 h and subsequently used to produce frozen sections. The DHE staining solution (100 μM) was added to the frozen sections and incubated at 37 °C for 30 min. Following DHE oxidation red fluorescence was emitted and the sections were viewed and photographed using a confocal laser-scanning microscope (Zeiss 710 microscope, Zeiss) (630×magnification).

### Hematoxylin–eosin (H&E) staining and evaluation of Cystitis

H&Estaining was applied to the paraffin sections according to routine protocols. All of the paraffin sections were assessed by a pathologist with experience in bladder pathology. The method of assessment mainly refers to the previous article of Bae WJ et al. [4] It is divided into four grades as follows: Grade 0 to Grade 3. Grade 0: morphologically unremarkable with slight or no inflammation or epithelial changes; Grade 1:mild inflammatory infiltrate in the lamina propria with scattered lymphocytes or monocytes accompanied by mild chronic edema, hemorrhage, or changes in urinary epithelium; Grade 2: moderate inflammatory infiltration of the lamina propria, with focal extension of inflammation to the lamina propria, accompanied by moderate chronic edema and hemorrhage; Grade 3: severe inflammation of the lamina propria and muscularis propria associated with urinary epithelial ulcers, severe chronic edema, hemorrhage and fibrin deposition.

### Analysis by RT-PCR

Total RNA was extracted and reverse-transcribed using kits as previously described [27]; quantitative PCR was performed using a LightCycler 480 Real-time PCR System to analyze the expression of pro-inflammatory mediators (*TNF-α*, *IL-1β*, and *IL-6*). The sequences of the primers (Sangon Bioengineering Co., Ltd., Shanghai, China) are listed in Table 1. The CT values were correlated with the initial DNA copy number. Relative gene expression was calculated using the double CT value method: △CT = CT_target gene_—CT_reference gene_ △△CT = △CT_target sample_—△CT_control_, where the relative expression level of the target genes = 2^−△△CT^, and the relative expression of the control group was 2^0^ = 1.

**Table 1:** Primers used in quantitative real-time PCR

Primers	Sequence (5′ → 3′)	
IL-1β	Forward	5′-TTGAGTCTGCACAGTTCCCC-3′
	Reverse	5′-TCCTGGGGAAGGCATTAGGA-3′
IL-6	Forward	5′-AGAGACTTCCAGCCAGTTGC-3′
	Reverse	5′-AGTCTCCTCTCCGGACTTGT-3′
TNF-α	Forward	5′-GGCTTTCGGAACTCACTGGA-3′
	Reverse	5′-GGGAACAGTCTGGGAAGCTC-3′
GAPDH	Forward	5′- AAGAGGGATGCTGCCCTTAC -3′
	Reverse	5′- ATCCGTTCACACCGACCTTC-3′

### Western blotting analysis

For WB analysis, we followed the methodology described previously [28]. The following primary antibodies were used: anti-phos-p65 (Ser536) antibody (dilution 1:1000), anti-p65 antibody (dilution 1:1000), anti-p38 antibody (dilution 1:1000), anti-phos-p38(Thr180/Tyr182) (dilution 1:1000), anti-IκBα antibody (dilution 1:1000), and GAPDH (dilution 1:10,000). Blot images were acquired on a Tanon 6600 luminous imaging workstation, and the optical density was analyzed using Image Pro Plus 6.0 software (Media Cybernetics, Rockville, MD, USA).

### Data analysis

Statistical analysis was performed using the SPSS software. All data were expressed as mean ± SD. *N* represented the number of animals. One-way ANOVA and Tukey’s test were used to evaluate the statistical significance of the differences between groups. *p* values of *p* < 0.05 (*) and *p* < 0.001(**) were considered for significant differences.

## Results

### Tt-SOD improves the pain threshold of chemical cystitis

**Fig. 1:**
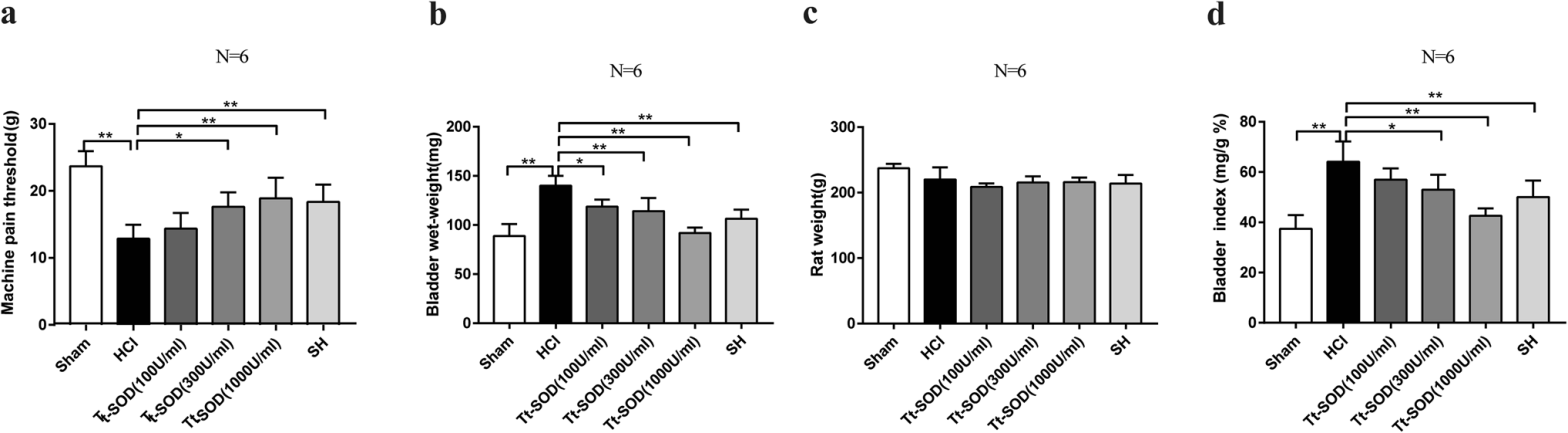
Tt-SOD treatment increases the mechanical threshold, decrease the wet weight of bladder, and decrease the bladder index in chemical cystitis rats. The machine pain threshold was assessed in the different groups (**a**). Assessment of the bladder wet weight (**b**), body weight (**c**) and bladder index in the different groups (**d**). The results are shown as mean ± SD (*N* = 6). *p < 0.05, **p < 0.01. *SH* Sodium hyaluronate; *Tt-SOD* Thermus thermophilic-superoxide dismutase

To assess whether Tt-SOD pretreatment alters the bladder-related pain on HCl-induced cystitis, we measured the perineal mechanical threshold as a substitute for bladder-related pain. The results indicated that the perineal mechanical threshold in the HCl group was significantly lower than that of the Sham group (*p* < 0.01), while that of the SH and Tt-SOD (300, 1000 U/ml) groups were significantly higher than those of the HCl group (*p* < 0.05) (Fig. 1a).

### Tt-SOD reduces the bladder index of chemical cystitis

The bladder index of each rat was calculated. As shown in Fig. 1b–d, the baldder wet weight was increased in the HCl group compared with Sham group (*p* < 0.05), while the Tt-SOD (100, 300, 1000 U/ml) and SH groups was decreased significantly compared with that of the HCl group; the body weight was not significantly different among the different groups (*P* > 0.05); the bladder index was increased in the HCl group compared with that of the Sham group (*P* < 0.01), while the Tt-SOD (300, 1000 U/ml) and SH groups was decreased significantly compared with that of the HCl group (*p* < 0.05).

### Tt-SOD lessens tissue damage and inflammatory cell infiltration

**Fig. 2:**
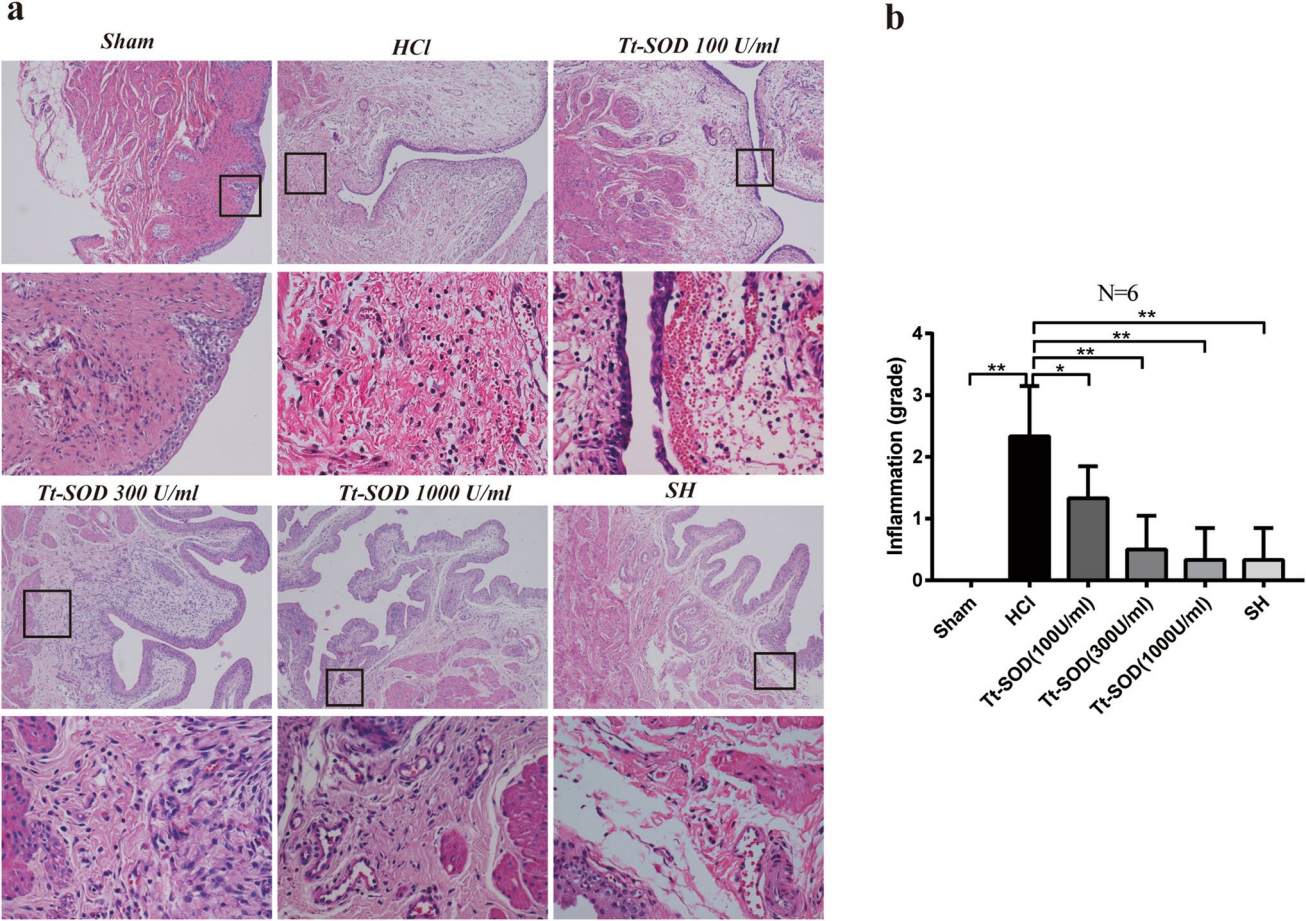
Tt-SOD treatment reduced inflammatory cell infiltration and tissue edema in bladder tissue sections. Representative H&E staining images (**a**) and the inflammation grade (**b**) of the rat bladder in each group. The results are shown as mean ± SD (*N* = 6). *p < 0.05, **p < 0.01. Lower panels show higher magnification images of insets in upper panels (□). (H&E; X100 and X400). *H&E* Hematoxylin&eosin; *SH* sodium hyaluronate; *Tt-SOD* Thermus thermophilic-superoxide dismutase

The effects of Tt-SOD on bladder tissues were investigated by H&E staining. The bladder tissue sections were observed under a light microscope (Olympus CKX53, Tokyo, Japan). The increased inflammatory cell infiltration, edema and urothelial exfoliation were identified on a bladder mucosa in the HCl group (Fig. 2a). The SH and Tt-SOD (100, 300, 1,000 U/ml) groups exhibited significantly decreased inflammatory cell infiltration and edema compared with those of the HCl group, and the inflammatory score was also decreased (*p* < 0.01) (Fig. 2b).

### Tt-SOD reduces serum levels of TNF-α, IL-1β, and IL-6

**Fig. 3:**
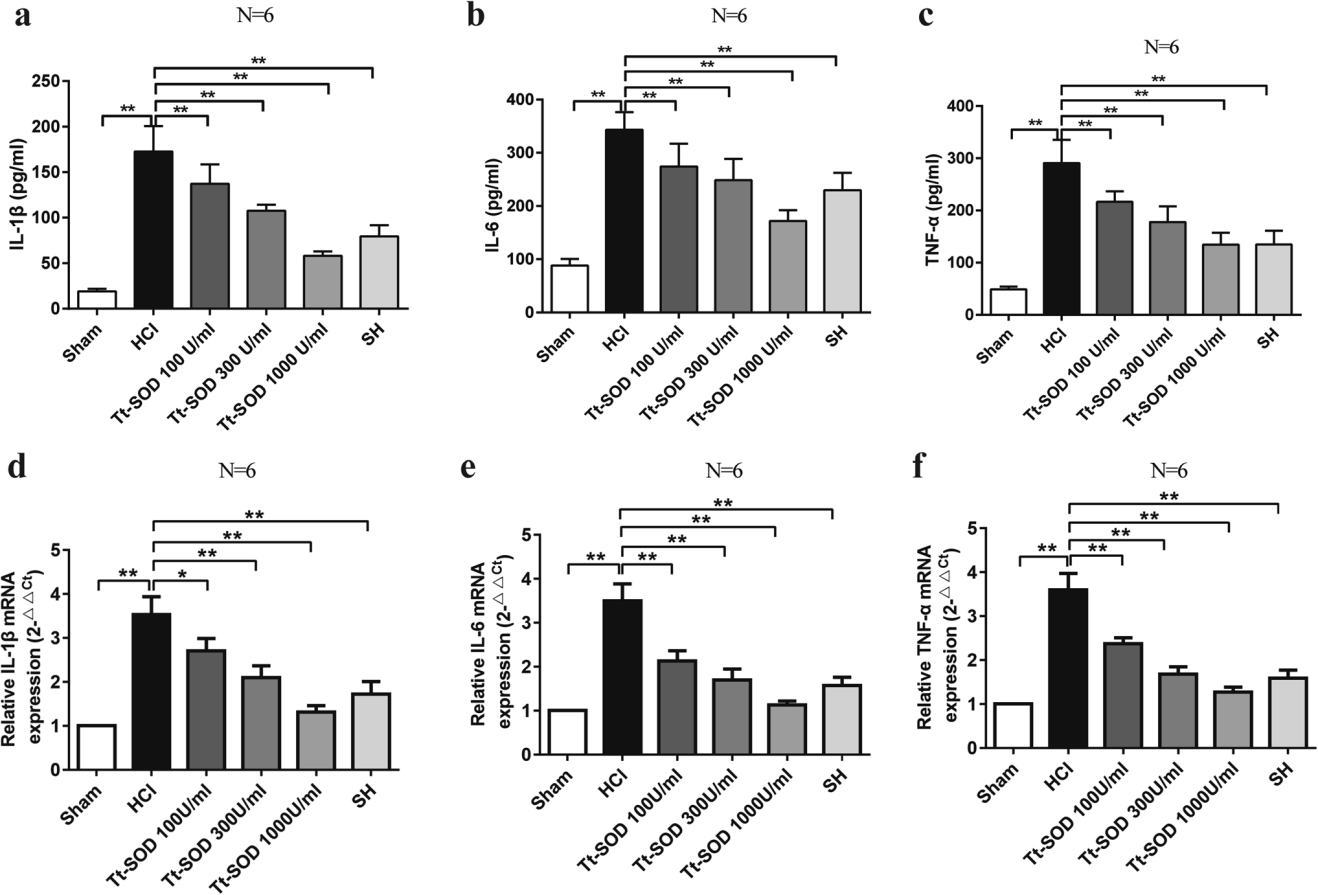
Tt-SOD treatment decreased the serum levels of inflammatory factors and the *mRNA* expression levels of inflammatory factors in bladder tissue. The expression levels of IL-1β (**a**), IL-6 (**b**), and TNF-α (**c**) from the indicated groups were measured by ELISA. The *mRNA* expression of *IL-1β* (**d**), *IL-6* (**e**), and *TNF-α* (**f**) were detected by RT-PCR. The results are shown as mean ± SD (*N* = 6). *p < 0.05, **p < 0.01. IL-1β, Interleukin-1β; IL-6, Interleukin-1; TNF-α, tumor necrosis factor-α; *mRNA* Messenger Ribonucleic Acid, *SH* Sodium hyaluronate; *Tt-SOD* Thermus thermophilic-superoxide dismutase

We assessed the therapeutic effects of Tt-SOD on HCl-induced cystitis by detecting the serum levels of certain inflammatory factors (TNF-α, IL-1β and IL-6) by specific Elisa kits. HCl treatment caused a significantly increased in the level of TNF-α, IL-1β and IL-6 as compared with control group. The SH and Tt-SOD (100, 300, 1000 U/ml) groups indicated a significant decrease in the level of TNF-α, IL-1β and IL-6 as compared with the HCl group (Fig. 3a–c).

### Tt-SOD reduces mRNA levels of TNF-α, IL-1β, and IL-6

To determine the effect of Tt-SOD on inflammatory cytokines, we detected the *mRNA* levels of *TNF-α*, *IL-1β*, and *IL-6* in the bladder tissue by real-time polymerase chain reaction (RT-PCR). HCl treatment caused a significantly increased in the *mRNA* level of *TNF-α, IL-1β and IL-6* as compared with Sham group. The SH and Tt-SOD (100, 300, 1000 U/ml) groups indicated a significant decrease in the *mRNA* level of *TNF-α, IL-1β and IL-6* as compared with the HCl group (Fig. 3d–f).

### Tt-SOD reduces ROS levels in bladder tissue

**Fig. 4:**
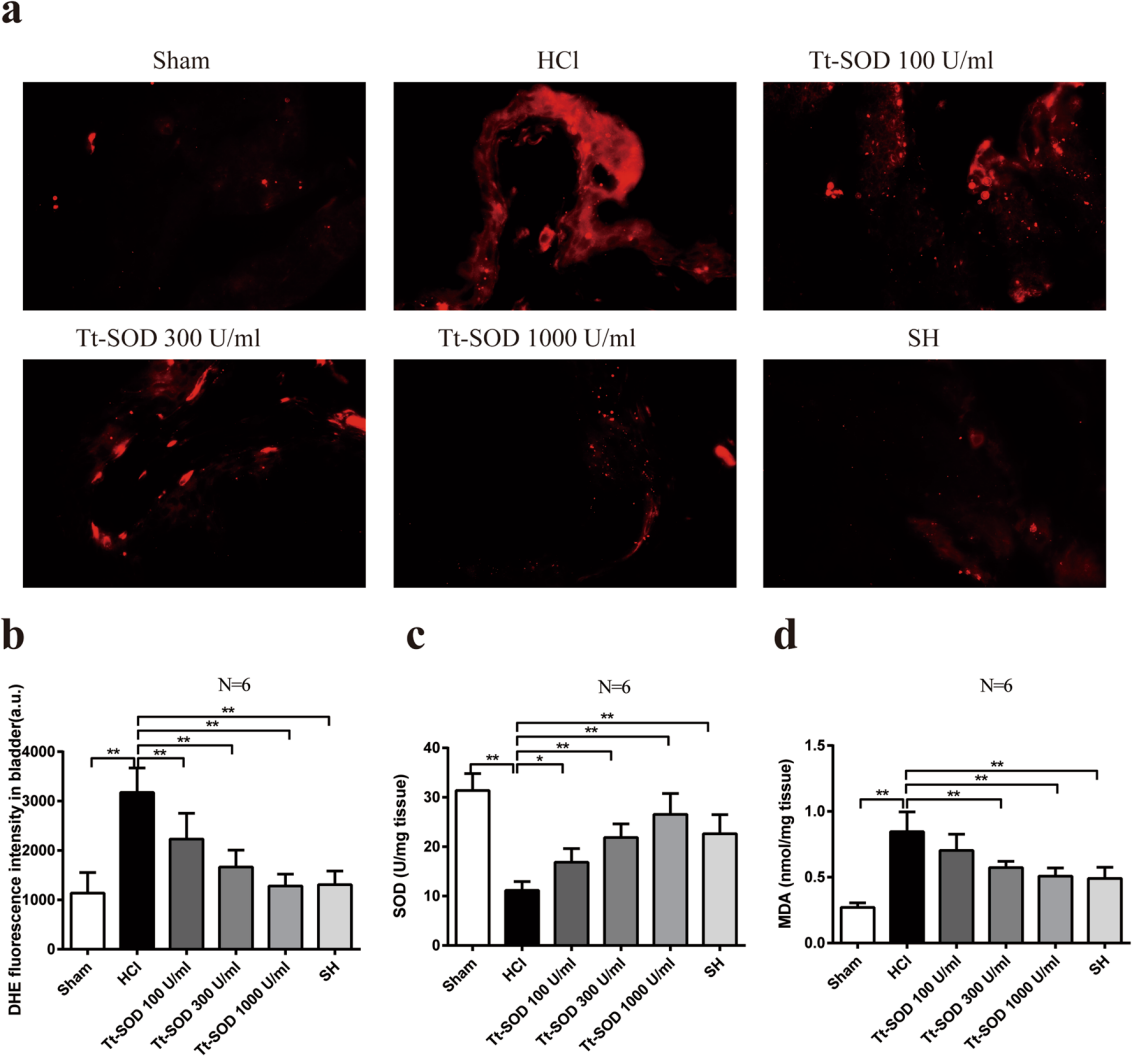
Tt-SOD exerts its antioxidant effects by decreasing the ROS and MDA levels and increasing the levels of SOD. Representative images of DHE staining (**a**) and quantification of DHE fluorescence intensity (red) (**b**). SOD activity (**c**) and MDA content (**d**) from the indicated group were detected by the SOD assay kit or the MDA assay kit. The results are shown as mean ± SD (*N* = 6). *p < 0.05, **p < 0.01. *DHE* Dihydroethidium; *ROS* reactive oxygen species; *SOD* superoxide dismutase; *MDA* malondialdehyde; *SH* sodium hyaluronate; *Tt-SOD* Thermus thermophilic-superoxide dismutase

To examine the effects of Tt-SOD on ROS production, the fluorescence intensity of the fluorescent probe, DHE, was evaluated. The results indicated that fluorescence intensity was substantially increased in the HCl group compared with that of the Sham group (*p* < 0.01), while it was significantly decreased in the SH and Tt-SOD groups at all doses compared to those of the HCl group (*p* < 0.01) (Fig. 4a, b).

### Tt-SOD increased SOD levels and reduced MDA levels

As shown in Fig. 4C, the SOD levels were significant decrease in the HCl group compared with that of the Sham group (*p* < 0.01), while the SH and Tt-SOD (100, 300, 1000 U/ml) groups exhibited a significant increase in SOD levels compared to those of the HCl group (*p* < 0.05). As shown in Fig. 4d, the MDA levels were significant increase in the HCl group compared with that of the Sham group (*p* < 0.01), while the SH and the Tt-SOD groups (300, 1000 U/ml) exhibited a significant decrease of MDA levels compared to those of the HCl group (*p* < 0.01). However, there was no difference identified between the Tt-SOD group (100 U/ml) and HCl group (*p* > 0.05).

### Tt-SOD inhibited the protein expression of NF-κB P65 and p38 MAPK signaling pathways

**Fig. 5:**
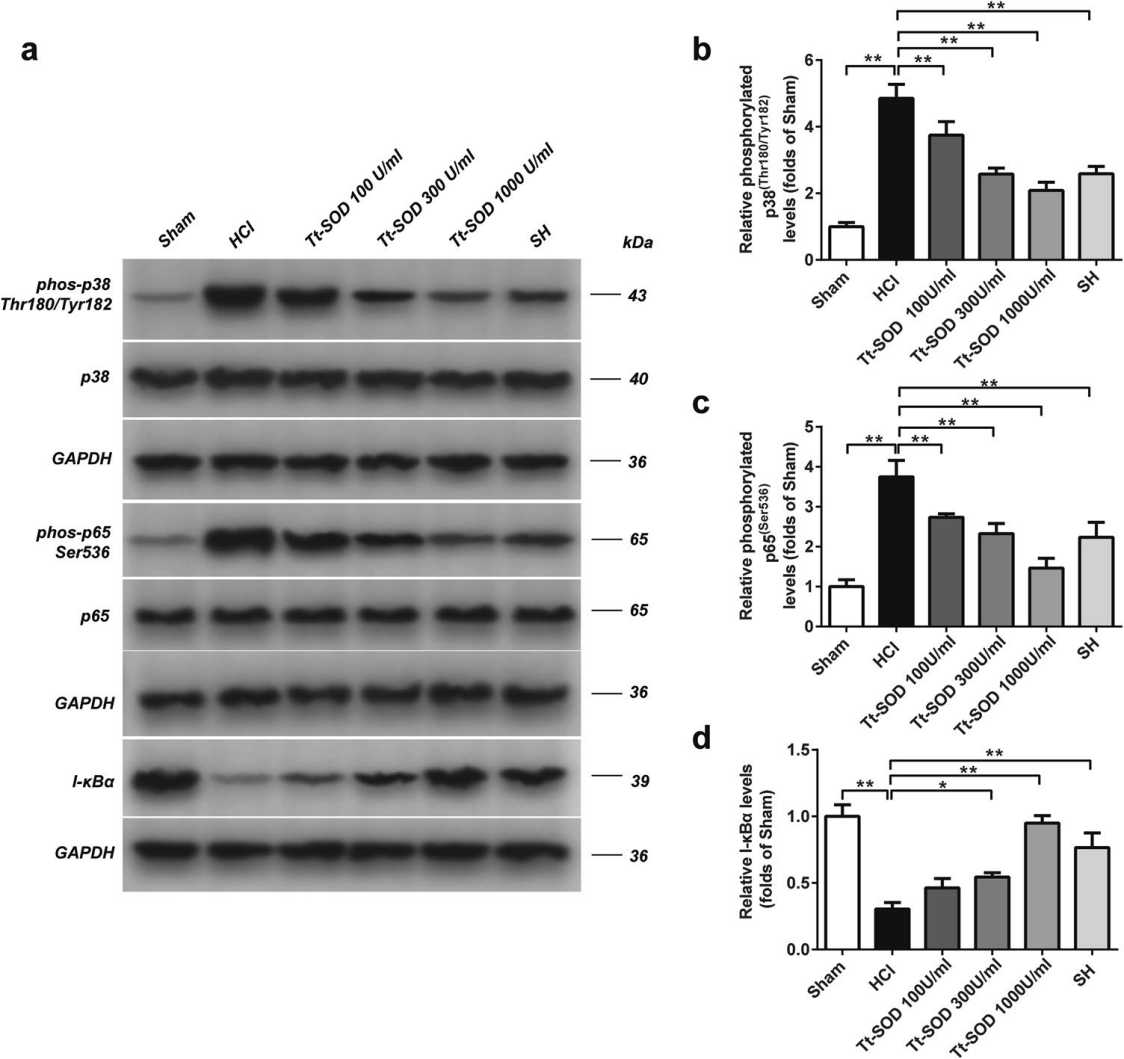
Tt-SOD exerts its anti-inflammatory and antioxidant through the NF-kB and p38 MAPK pathway in the chemical cystitis model. Western blotting analysis for phos-p65 Ser536, p65, phos-p38 Thr180/Tyr182, p38, I-κBα and GAPDH (**a**). Densitometric analysis of phos-p38 Thr180/Tyr182 (**b**), phos-p65 Ser536 (**c**) and I-κBα (**d**). The WB is a representative image of an experiment. *MAPK* Mitogen-activated protein kinase; *I-κBα* Inhibitor of nuclear factor-κBα; *NF-κB* nuclear factor-κB; *GAPDH* glyceraldehyde-3-phosphate dehydrogenase; *SH* sodium hyaluronate; *Tt-SOD* Thermus thermophilic-superoxide dismutase

To determine the therapeutic mechanism of Tt-SOD on chemical cystitis, we examined the expression of phosphorylated p65, p65, phosphorylated p38, p38, and I-κBα proteins in the bladder tissue. As shown in Fig. 5a–d, P65 and P38 phosphorylation levels were significantly increased, and I-κBα levels significantly decreased, in the HCl group compared with those in the Sham group (*p* < 0.01). Compared with those in the HCl group, P65 and P38 phosphorylation levels were significantly decreased, and I-κBα levels significantly increased (*p* < 0.05), in the SH group and Tt-SOD groups (300, 1000 U/ml).

## Discussion

Cystitis in humans is characterized by the symptoms of urinary frequency, urgency and pain at voiding [29]. This disease cannot be treated adequately currently, due to the variety of sources and the wide range of symptoms. At present, animal models of cystitis are mainly generated by injecting toxic irritants into the bladder of the animals. The toxic irritants include hydrochloric acid, acetic acid, acetone, allyl aldehyde, lipopolysaccharide, protamine sulfate and cyclophosphamide, among which hydrochloric acid is a relatively simple and stable agent that can be used to induce chemical cystitis [22, 30]. Previous studies have demonstrated that HCl-induced chemical cystitis in SD rats causes apparent bladder mucosal injury, increased urination time and increased bladder-related pain [23]. In this study, Tt-SOD can increase the pain threshold, and reduce the bladder tissue edema, the inflammatory cell infiltration, and the serum inflammatory cytokine levels, as well as inhibit the expression of inflammatory factors. We further investigated the mechanism underlying the anti-inflammatory action of Tt-SOD notably in conjunction with the disruption of the balance of free radical generation and the regulation of the antioxidant enzyme system that can affect the induction of oxidative stress [31].

Various experiments have shown that SOD plays an important role in various biological functions, such as ROS scavenging [32–34]. Kanai et al. [35] selectively induced the production of Mn-SOD in the bladder mucosal epithelia of rats treated by radiation and subjected to gene therapy. This resulted in the significant reduction of the ROS levels of the bladder mucosa and the restoration of the normal bladder mucosal function. Moreover, Murphy et al. [36] reported that treatment of SOD M40403 caused a significant decrease in the ROS levels and the degree of inflammation noted in oral mucositis. Bayrak et al. [37] also studied the effects of intravesical dexpanthenol use on bladder histology and lipidperoxidation in a model of chemical cystitis. They found that difentanyl alleviated the treatment of chemical cystitis, had anti-inflammatory effects, promoted bladder epithelial growth, and reduced MDA levels in tissue and serum. These previous findings correspond with our results. Compared with untreated cystitis (HCl group), treatment with the Tt-SOD increased SOD activity, decreased the MDA and ROS levels. It is well known that the NF-κB P65 and P38 MAPK pathways are involved in inflammatory and oxidative stress responses [38–40]. Our results indicated that Tt-SOD has anti-inflammatory and anti-oxidative stress effects by inhibiting the NF-κB P65 and P38 MAPK pathways, reducing oxidative stress and inflammation. P38 MAPK regulates downstream transcription factors after activation by ROS, a hyperosmotic state, or cytokines. The transcription of inflammatory factors depends on the activation of NF-κB, and the activity of NF-κB is reduced by its inhibitor, I-κBα [41]. Notably, to confirm the efficacy of the Tt-SOD, we compared its efficacy with the known SH used as a positive control. The findings showed that Tt-SOD have effect on chemical cystitis in a concentration-dependent manner, with the highest concentration (1000 U/ml) having the most efficacy and even better efficacy than the SH group (positive control).

Hyaluronic acid (HA) is an acidic mucopolysaccharide that is involved in inflammation, angiogenesis, fibrosis, cancer, oxidative stress, and other processes. SH is an extremely stable form of HA, which is often used as a protective agent for the bladder mucosa in cystitis. Therefore, SH was used as the positive control [42]. When existing antioxidant defense mechanisms fail to regulate excessive ROS production, cells enter a state of oxidative stress that disrupts the redox balance. This activates the NF-κB and MAPK signaling pathways, leading to the production of inflammatory cytokines [42, 43]. Inflammation stimulates further production of ROS, creating a continuous adverse cycle. Inflammatory cytokines and ROS are involved in the occurrence, development, and progression of chemical cystitis. HA with a molecular weight of < 500 kDa induces inflammation; however, a high molecular weight (> 500 kDa) HA has anti-inflammatory effects. SH is a high molecular weight polymer (500–730 kDa) with anti-inflammatory effects. In addition, HA regulates AKT phosphorylation via nuclear factor erythroid 2-related factor 2 (NRF2), resulting in ROS reduction and antioxidant effects [44]. In most rat cystitis models, SH is used as a bladder mucosal protective agent because of its anti-inflammatory and antioxidant properties. SH is generally considered safe and is well tolerated; however, immune responses, such as late-onset allergies and a granulomatous reaction, have been reported [45, 46]. Compared with SH, SOD generally does not cause immune responses, and local injections of SOD are already used in some countries to relieve sports injuries, osteoarthritis, and other inflammatory conditions [47]. Side effects of SOD are rarely reported, and only occasional local pain may occur after intramuscular injection. Tt-SOD that was used in this study overcomes the limitations of SOD, such as the instability and low or even no activity in the process of administration and easy degradation and inactivation in the digestive tract. Tt-SOD shows superior stability and activity under high temperature, highly acidic and alkaline conditions, and in the presence of various proteases, such as pepsin and trypsin. Tt-SOD also exhibits good anti-inflammatory and antioxidant effects against bladder inflammation. Therefore, Tt-SOD is very promising as a therapeutic agent for chemical cystitis.

The present study suggests, for the first time, that treatment with Tt-SOD have anti-inflammatory and decrease antioxidant stress in the rat model of chemical cystitis induced by hydrochloric acid. NF-κB p65 and p38 MAPK signaling pathways play key roles in the ability of Tt-SOD to regulate inflammatory responses and oxidative stress injury in the rat model of chemical cystitis. These results suggest that Tt-SOD is useful for treating chemical cystitis. The detail mechanisms of Tt-SOD in chemical cystitis will be further investigated in the future, and our further work is to observe the therapeutic effect of Tt-SOD in chemical cystitis model caused by bladder cancer.

## Conclusions

In this study, the HCl-induced model of chemical cystitis reduced inflammation and promoted the repair of bladder mucosa injury in rats after Tt-SOD treatment, which may be related to the amelioration of oxidative stress and inflammation by Tt-SOD treatment. Therefore, further experimental and clinical studies are needed to provide more data to support the role of Tt-SOD in the treatment of chemical cystitis, and Tt-SOD may shows promised for used as an adjuvant drug to reduce the occurrence of chemical cystitis during intravesical chemotherapy for bladder cancer in future.

## Abbreviations

SOD: Superoxide dismutase
Mn: Manganese
HCl: Hydrochloric acid
MDA: Malondialdehyde
SH: Sodium hyaluronate
Elisa: Enzyme-linked immunosorbent assay
DHE: Dihydroethidium
MPO: Myeloperoxidase
IBD: Inflammatory bowel disease
HE: Hematoxylin–eosin
ROS: Reactive oxygen species
a.u.: Arbitrary unit
